# E2F transcription factor 1 elevates cyclin D1 expression by suppressing transcription of microRNA‐107 to augment progression of glioma

**DOI:** 10.1002/brb3.2399

**Published:** 2021-11-10

**Authors:** Huan Xie, Shigang Lv, Zhaozhen Wang, Xinzhang Yuan

**Affiliations:** ^1^ Department of Clinical Medicine Jiangxi Health Vocational College Nanchang P. R. China; ^2^ Department of Neurosurgery The Second Affiliated Hospital of Nanchang University Nanchang P. R. China

**Keywords:** cyclin D1, E2F transcription factor 1, glioma, miR‐107, Wnt/β‐catenin

## Abstract

**Background:**

Dysregulation of microRNAs has been frequently implicated in the progression of human diseases, including glioma. This study aims to explore the interaction between E2F transcription factor 1 (E2F1) and miR‐107 in the progression of glioma.

**Methods:**

Expression of miR‐107 in glioma tissues and cells was examined. Putative binding sites between E2F1 and the promoter region of miR‐107, and between miR‐107 and cyclin D1 (CCND1) mRNA were predicted via bioinformatic systems and validated via chromatin immunoprecipitation and luciferase reporter gene assays. Altered expression of miR‐107, E2F1, and CCND1 was introduced in A172 and T98G cells to examine their roles in cell growth and the activity of the Wnt/β‐catenin signaling. In vivo experiments were performed by injecting cells in nude mice.

**Results:**

miR‐107 was poorly expressed, whereas E2F1 and CCND1 were highly expressed in glioma tissues and cells. E2F1 bound to the promoter region of miR‐107 to induce transcriptional repression, and miR‐107 directly bound to CCND1 mRNA to reduce its expression. Overexpression of miR‐107 reduced proliferation, migration and invasion, and augmented apoptosis of glioma cells, and it reduced activity of the Wnt/β‐catenin pathway. The anti‐tumorigenic roles of miR‐107 were blocked by E2F1 or CCND1 overexpression. Similar results were reproduced in vivo where miR‐107 overexpression or E2F1 inhibition blocked tumor growth in nude mice.

**Conclusion:**

This study suggested that E2F1 reduces miR‐107 transcription to induce CCND1 upregulation, which leads to progression of glioma via Wnt/β‐catenin signaling activation.

## Graphical abstract

By performing cellular and animal experiments, we confirmed that the transcription factor E2P1 can induce transcriptional repression of miR‐107 and block its inhibitory effect on CCND1, which leads to the malignant development of glioma with the involvement of the Wnt/β‐catenin signaling pathway. This study confirmed the oncogenic roles of E2F1 and CCND1 and the anti‐tumorigenic role of miR‐107 in glioma.

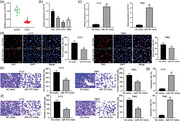

## 1 INTRODUCTION

Glioma is the most frequent and aggressive type of primary tumor of the central nervous system (CNS) and a major cause of brain cancer‐related deaths (Chen et al., 2017; Ludwig & Kornblum, 2017). According to World Health Organization (WHO) classification, there are four grades of gliomas (grades I–IV): grade I and II are non‐malignant tumors, grade III tumors are malignant and grade IV tumors, termed glioblastomas (GBMs), are the most malignant and fatal (Louis et al., 2016; Tamtaji et al., 2020). Current clinical treatments for glioma mainly include surgery, radiotherapy, adjuvant chemotherapy, targeted therapy, and immunotherapy (Weller et al., 2013). However, prognosis of patients with glioma, especially for those with GBM, remains unfavorable, and the median survival time of these patients is 15–23 months (Ostrom et al., 2014; Shergalis et al., 2018). There has been an urgent need for the development of more effective therapeutic options for glioma.

The molecular profiling of cancer has arisen due to the clinical values of key molecules in diagnosis, prognosis, and therapy of patients (Diamandis & Aldape, 2017). MicroRNAs (miRNAs) are a subclass of short non‐coding RNAs approximately 22 nucleotides long and they are emerging molecules in the field of cancer research owing to their involvements in fundamental cellular processes and potent regulation on target mRNAs (Harrandah et al., 2018). The prognostic and diagnostic values of miRNAs in glioma have aroused increasing concerns (Zhou et al., 2018). miR‐107 has been demonstrated as a candidate tumor suppressor in several human malignancies such as colorectal cancer (Fu et al., 2019) and cervical cancer (Li et al., 2019). Moreover, miR‐107 has been reported to be poorly expressed in glioma cells (Zhen et al., 2019), and downregulation of miR‐107 was correlated with invasiveness, proliferation, and stem‐like properties of glioma cells (Wu et al., 2020; Yang et al., 2017). This body of evidence suggested that miR‐107 may serve as a tumor suppressor in glioma. E2F transcription factor 1 (E2F1) is a member of the E2Fs family of transcription factors that mediate transcription activity of genes implicated in development, differentiation, proliferation, and apoptosis (Muller et al., 2001). Downregulation of E2F1 by miRNAs has been observed to be associated with reduced proliferation of glioma cells (Huang & Chi, 2019; Xia et al., 2019). Interestingly, miR‐107 was found to bind to E2F1 mRNA to regulate its expression (Carroll et al., 2012). Moreover, in addition to serving as miRNA targets, as a transcription factor, E2F1 can also regulate the transcription activity of specific transcripts including miRNA (Aguilar et al., 2021; Li et al., 2019). The integrated bioinformatic analyses in the present study suggested that E2F1 had a potential binding relationship with the promoter region of miR‐107 which in turn had a binding sequence with the 3ʹuntranslated region (3ʹUTR) of cyclin D1 (CCND1) mRNA. CCND1 has recently been revealed to be highly expressed in glioma and reduced cancer cell apoptosis (Sun et al., 2020). Therefore, this study hypothesized that there might be an E2F1/miR‐107/CCND1 axis involved in the pathogenesis of glioma. Altered expression of these molecules was induced in glioma cells for in vitro and in vivo experiments to validate this hypothesis.

## 2 MATERIALS AND METHODS

### 2.1 Ethical approval

This research was approved by the Ethics Committee of the Second Affiliated Hospital of Nanchang University and was performed in compliance with the Declaration of Helsinki. All eligible participants signed a written informed consent. The animal experiments adhered to the Guide for the Care and Use of Laboratory Animals (NIH, Bethesda, Maryland, USA).

### 2.2 Sample collection

Glioma tissue samples were obtained from the neurosurgery department of the Second Affiliated Hospital of Nanchang University. Samples from 23 patients with glioma treated from January 2018 to August 2019 were included. All samples were confirmed as glioma tissues by pathological examination. Another 10 normal brain tissues collected from patients who underwent intracranial surgery for craniocerebral injury were collected as control samples. All tissue samples were instantly frozen in liquid nitrogen and stored at −80℃ until further use.

### 2.3 Immunochemistry

The tissue samples were embedded in paraffin, cut into 5‐μm sections, dewaxed in xylene, and rehydrated in alcohol. After treatment with citrate buffer for antigen retrieval and treatment with 1% bovine serum albumin for 1 h, the sections were hybridized with anti‐CCND1 (1:200; Abcam Cat: ab16663; RRID: AB_443423; Abcam Inc., Cambridge, MA, USA) at 4℃ overnight and then incubated with horseradish peroxidase (HRP)‐labeled immunoglobulin G (IgG, 1:1,000; Abcam Cat#: ab6721; RRID: AB_955447; Abcam Inc) at 25℃ for 1 h. The staining was developed by 3,3′‐diaminobenzidine (Boster Biological Technology Co., Ltd., Wuhan, Hubei, China). The nuclei were stained by hematoxylin (Servicebio, Wuhan, Hubei, China). After that, the section slides were sealed and observed under a microscope.

### 2.4 Cell culture and treatment

A normal brain glial cell line Heb was procured from Jennio‐bio Co., Ltd (Guangzhou, Guangdong, China), glioma cell lines U251 and A172 were procured from Cell Bank of Chinese Academy of Sciences (Shanghai, China), and glioma T98G cells were procured from American Type Culture Collection (Manassas, VA, USA). All cells were cultured in high‐glucose Dulbecco's modified Eagle's medium supplemented with 10% fetal bovine serum (FBS, Gibco Company, Grand Island, NY, USA) at 37℃ with 5% CO_2_. After adherence, the cells were digested in 0.25% trypsin (Hyclone Company, Logan, UT, USA).

The lentivirus (LV) packaging systems including LV5‐GFP (carrying gene overexpressing plasmid) and pGLVU6/GFP [carrying short hairpin RNA (shRNA)] were procured from Genechem Co., Ltd. (Shanghai, China). Negative control (NC)‐mimic, miR‐107 mimic, shRNA of E2F1 (sh‐E2F1), overexpressing vector of E2F1 (oe‐E2F1) and CCND1 (oe‐CCND1) were provided by GenePharma Co., Ltd. (Shanghai, China). The LV packages‐carried target plasmids were transfected into HEK293T cells. According to the transfection combinations, the cells were allocated into NC mimic group, miR‐107 mimic group, miR‐107 + oe‐NC group, miR‐107 + oe‐E2F1 group, and sh‐E2F1 + oe‐CCND1 group. In detail, glioma cells in good growth condition were digested in trypsin and resuspended to 5 × 10^4^ cells/ml. The cell suspension was cultured in 6‐well plates (2 ml per well) at 37℃ overnight. After that, the cells in each well were transfected with the LV packages‐carried target plasmids in 1 ml culture medium (MOI: 5–10). Interference of two target genes was achieved by infecting cells with two LV packages concomitantly. After 48 h, the supernatant was collected. The LV particles in the supernatant were diluted, and the virus titer was examined. Exponentially growing virus was collected. Stably transfected cell colonies were screened by puromycin resistance, and the stable transfection was further validated by enzyme digestion and electrophoresis, reverse transcription‐quantitative polymerase chain reaction (RT‐qPCR), and sequencing.

### 2.5 Chromatin immunoprecipitation (ChIP)‐qPCR

The glioma cells were fixed in methanol for 10 min for DNA‐protein crosslinking. The cells were fractured via ultrasonication to obtain chromatin fragments. The fragments were centrifuged at 4℃ for 10 min at 12,000 rpm to collect the supernatant into two tubes. The tube was incubated with anti‐IgG (negative control) (1:2500; Abcam Cat#: ab6785; RRID: AB_955241; Abcam Inc. ) or anti‐E2F1 (1:1000, Abcam Cat#: ab112580; RRID: none; Abcam Inc.) at 4℃ overnight. The DNA‐protein complex was precipitated using Protein A agarose and was centrifuged for 5 min at 12,000 rpm to discard the supernatant. The non‐specific binding complexes were washed away, and the specific binding ones were de‐crosslinked at 65℃ overnight. The DNA fragments were extracted using phenol/chloroform, purified, and collected. Enriched fragments in the miR‐107 promoter were examined by qPCR.

### 2.6 TOP/FOP flash assay

The wild‐type (TOP) and mutant‐type (FOP) LEF/TCF reporter genes were cloned to the pGL3 luciferase constructs (Promega Corp., Madison, WI, USA). Cells in each group were cultured in 48‐well plates at a density of 2 × 10^4^ cells per well for 24 h. Each well was loaded with 0.2 μg TOP/FOP flash and 1 ng Renilla (pRLTK) luciferase‐encoding plasmid (Promega) according to the instructions of a Lipofectamine 3000 kit (Thermo Fisher Scientific Inc., Waltham, MA, USA). After 24 h, the luciferase intensity and Renilla signals were detected using a dual‐luciferase reporter gene kit (Promega) according to the kit's instructions. The calculation formula was as follows: TOP‐flash/FOP‐flash (Liu et al., 2021; Lu et al., 2018).

### 2.7 Immunofluorescence staining

The glioma cells were seeded on slides and fixed with 4% paraformaldehyde (PFA) for 10 min. Thereafter, the cells were incubated with anti‐β‐catenin (1:200; Abcam Cat#: ab223075; RRID: none; Abcam Inc.) at 4℃ overnight and warm‐incubated with Alexa Fluor® 647‐conjugated secondary antibody (1:500; Abcam Cat#: ab150075; RRID: AB_2752244; Abcam Inc.) for 1 h. The nuclei were stained with Hoechst. The images were captured under an inverted microscope (Ti2‐E, Nikon Instruments Inc., Tokyo, Japan).

### 2.8 5‐ethynyl‐2′‐deoxyuridine (EdU) labeling

The glioma cells were cultured in 24‐well plates for 48 h. Each well was loaded with 300 μl renewed culture medium containing 5‐ethynyl‐2′‐deoxyuridine (EdU) solution (1:1000) for 2 h. The cells were fixed with 4% PFA for 15 min, washed in phosphate‐buffered saline (PBS), and permeabilized for 10 min at room temperature. Next, the cells in each well were added with 300 μl green‐fluorescence (555 nm) EdU reaction mixture (C10341‐3, RiboBio Co., Ltd., Guangzhou, Guangdong China) for 30 min of warm incubation at 25℃. The nuclei were stained with Hoechst solution (C1026, Beyotime Biotechnology Co., Ltd., Shanghai, China) in the dark at 25℃ for 15 min. After two PBS washes, the staining was observed under an inverted microscope.

### 2.9 Transwell assay

Cell migration and invasion were examined using transwell chambers (8 μm, Corning Glass Works, Corning, NY, USA). The transfected cells were resuspended in serum‐free medium and were sorted into the apical chambers. The basolateral chambers were loaded with 600 μl 20% FBS‐contained medium. For cell invasion measurement, the apical chambers additionally were pre‐coated with 50 μl Matrigel (BD Biosciences, Franklin Lakes, NJ, USA). After incubation at 37°C for 48 h, cells migrated or invaded to the basolateral chambers were fixed with 4% PFA and stained with 0.1% crystal violet. The number of migrating and invading cells was counted under a Nikon microscope (×200) with five representative fields of views included.

### 2.10 Flow cytometry

An Annexin V‐phycoerythrin (PE)/7‐aminoactinomycin D (7‐AAD) kit (BD Biosciences) was used to examine cell apoptosis. The 7‐AAD is a standard flow cytometry probe used to distinguish living and non‐living cells. In short, the glioma cells were resuspended in 1 × binding buffer and incubated with Annexin V‐PE/7‐AAD in the dark at 25℃ for 15 min. Apoptosis of cells was then analyzed using a FACS Canto™II flow cytometer (BD Biosciences) within 1 h.

### 2.11 Dual luciferase reporter gene assay

The specific binding sequence between miR‐107 and CCND1 mRNA was predicted from the bioinformatic system LNCIPEDIA (https://lncipedia.org/), and the binding relationship was validated by a luciferase assay. In brief, the whole blood genomic DNA was extracted and used as the template. The wild‐type (WT) CCND1‐3′UTR‐WT fragment containing the putative binding sequence was PCR amplified. After DNA retrieval and purification, the target fragments were inserted into the dual luciferase reporter gene vectors. After translational amplification, the monoclon was selected for colony PCR, and the vectors were extracted and identified by double enzyme digestion and DNA sequencing. The mutant‐type (MUT) sequence CCND1‐3′UTR‐MUT was constructed by designing mutation fragments on the WT sites. The CCND1‐3′UTR‐MUT no longer had binding relationship with miR‐107. The CCND1‐3′UTR‐WT and CCND1 3′UTR‐MUT sequences were inserted into pmirGLO vectors (VT1439, BioVector Science Lab., Inc., Chongqing, China). It was confirmed that no other mutations were introduced. Thereafter, the CCND1 3′UTR‐WT and CCND1 3′UTR‐MUT luciferase vectors were co‐transfected with NC mimic or miR‐107 mimic into 293T cells utilizing a Lipofectamine™ 2000 kit (11668019, Thermo Fisher Scientific) according to the instruction manual. After 24 h, the luciferase activity in the cells was examined using a Dual‐Luciferase Reporter Assay Kit (Promega).

### 2.12 RT‐qPCR

Total RNA from tissues or cells was isolated using an RNA isolation kit (QIAGEN, Qiagen GmbH, Hilden, Germany). mRNA wasreverse‐transcribed to cDNA using a PrimeScript RT reagent Kit (RR047A, Takara Holdings Inc., Kyoto, Japan), whereas miRNA was reverse‐transcribed to cDNA with a miRNA First Strand cDNA Synthesis (Tailing Reaction) kit (B532451‐0020, Sangon Biotech Co., Ltd., Shanghai, China). Next, real‐time qPCR was conducted using a Fast SYBR Green PCR (Applied Biosystems, Foster, CA, USA) on an ABI 7500 real‐time PCR System (Applied Biosystems, Inc., Carlsbad, CA, USA). The primers are listed in Table 1, in which glyceraldehyde‐3‐phosphate dehydrogenase (GAPDH) and U6 were used as the endogenous controls. Relative gene expression was evaluated using the 2^–ΔΔCt^ method.

**TABLE 1:** Primer sequences

Primers	Sequence (5′−3′)
miR‐107	F: AGCAGCATTGTACAGGGCTATCA
	R: ATTGCGTGTCGTGGAGTCG
E2F1	F: TGCTCTCCGAGGACACTGACAG
	R: TCTTGCTCCAGGCTGAGTAGAGAC
CCND1	F: ATTCTTAATGCTTCCGTCTCTC
	R: GAGAGACGGAAGCATTAAGAAT
GAPDH	F: ATGGGGAAGGTGAAGGTCG
	R: GGGGTCATTGATGGCAACAATA
U6	F: GCGCGTCGTGAAGCGTTC
	R: GTGCAGGGTCCGAGGT

*Abbreviations*: miR‐107, microRNA‐107; E2F1, E2F transcription factor 1; CCND1, cyclin D1; GAPDH, glyceraldehyde‐3‐phosphate dehydrogenase.

### 2.13 Western blot analysis

Cells were lysed in protease‐ and phosphatase‐inhibitors‐supplemented radio‐immunoprecipitation assay (RIPA) cell lysis buffer (R0010, Solarbio Science & Technology Co., Ltd., Beijing, China) to extract total protein. After protein concentration determination using a BCA kit (ab102536, Abcam), an equal amount of protein sample (50 μg) was separated by 10% SDS‐PAGE and transferred onto nitrocellulose membranes (Beyotime). After being blocked in 5% non‐fat milk and blocked in 0.1% Tween‐20, the membranes were co‐incubated with anti‐E2F1 (1:1,000; Abcam Cat#: ab112580; RRID: none; Abcam Inc.), anti‐CCND1 (1:1,000; Abcam Cat#: ab16663; RRID: AB_443423; Abcam Inc.), anti‐β‐catenin (1:1,000, Abcam Cat#: ab223075; RRID: none; Abcam Inc.), anti‐Wnt10B (1:1,000; Abcam Cat#: ab70816; RRID: AB_1271486; Abcam Inc.), and anti‐GAPDH (1:2,500; Abcam Cat#: ab9485; RRID: AB_307275; Abcam Inc.) at 4℃ overnight. The next day, the membranes were incubated with HRP‐labeled secondary antibody IgG (1:20,000; Abcam Cat# ab205718; RRID: AB_2819160; Abcam Inc.) at 25℃ for 1 h. The protein blots were developed using the enhanced chemiluminescence reagent (BB‐3501, Amersham Biosciences, Uppsala, Sweden) and were analyzed using an image scanner (Amersham Biosciences). Protein level (relative to GAPDH) was examined using Image J software.

### 2.14 Xenograft tumors in nude mice

A172 cells stably transfected with NC mimic, miR‐107 mimic, sh‐NC + oe‐NC, sh‐E2F1 + oe‐NC, sh‐E2F1 + oe‐CCND1 were resuspended in serum‐free medium to 2 × 10^6^ cells/ml. Twenty‐five specific pathogen‐free grade nude mice (4–6 weeks old, 17–20 g) procured from SLAC Laboratory Animal Co., Ltd. (Shanghai, China) were allocated into five groups, *n* = 5 in each. After animal anesthesia using diethyl ether and disinfection, 2 × 10^5^ A172 cells (100 μl) with above transfections were injected into the right hemisphere of the mice via stereotactic injection. In short, the mice were anaesthetized by 80 mg/kg pentobarbital sodium (1%). The head was fixed, and the disinfected injection needle with cell suspension was placed in the surgical area above the mouse skull. An injection hole was drilled, and the injection site was located 0.62 mm in front of the bregma, 1.5 mm to the right of the midline, and 3.5 mm below the dura. The needle was placed into the injection hole, and the cell suspension was slowly injected into the brain tissue. After injection, the needle was taken out, and the wound was disinfected and glued with bone wax. On the 21st day after injection, the animals were euthanized by intraperitoneal injection of 150 mg/kg pentobarbital sodium. The volume of the xenograft tumors was calculated as follows: volume = length × width^2^/2, and the tumor weight was examined.

### 2.15 Statistical analysis

SPSS21.0 (IBM Corp. Armonk, NY, USA) was applied for data analysis. Measurement data were shown as the mean ± standard deviation (SD) from three independent experiments. The unpaired *t‐*test was applied for comparison between every two groups. Differences among multiple groups were compared by the one‐ or two‐way analysis of variance (ANOVA). *p* < .05 indicated that the difference was statistically significant.

## 3 RESULTS

### 3.1 miR‐107 inhibits proliferation, migration and invasion, and promotes apoptosis of glioma cells

**FIGURE 1:**
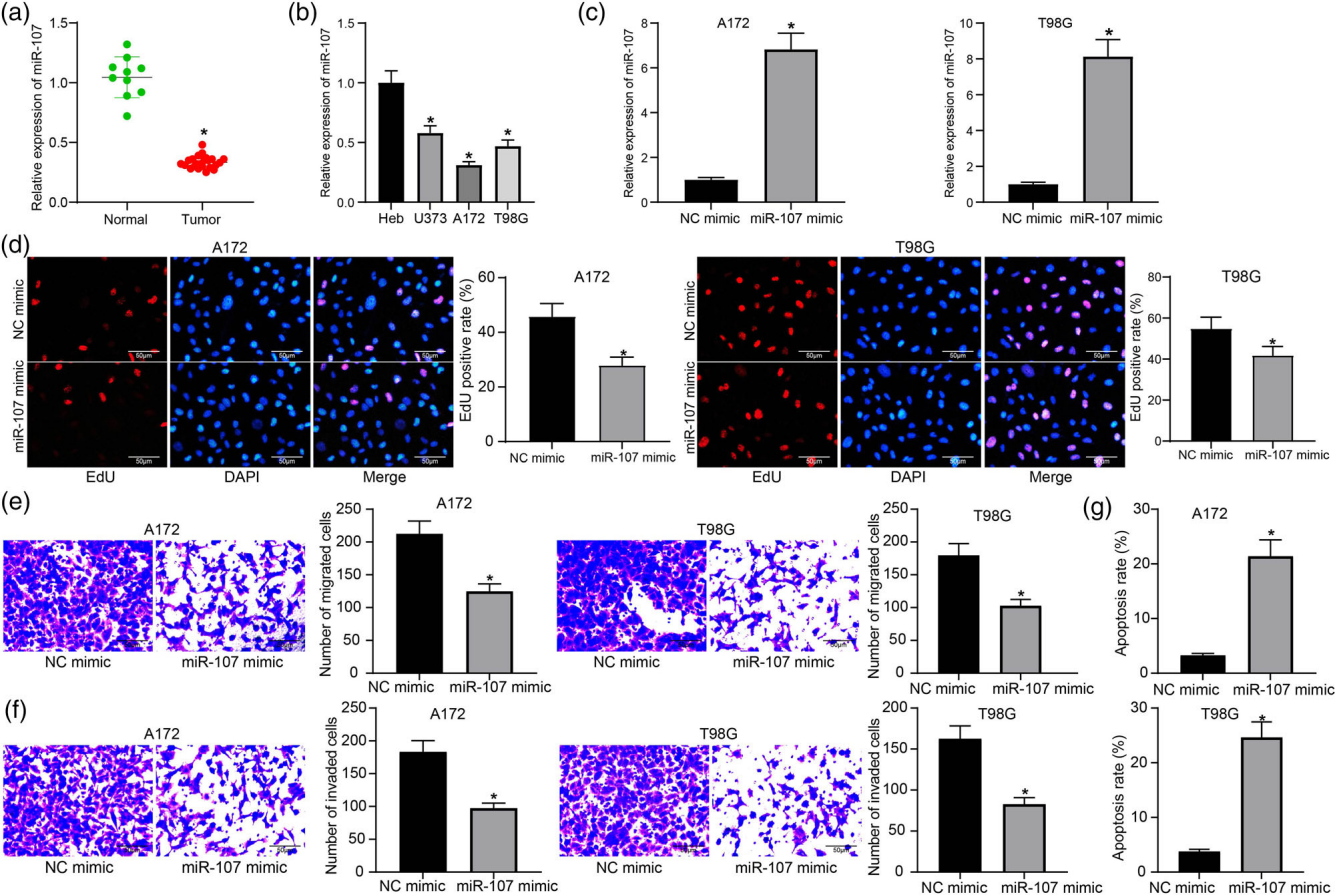
Overexpression of miR‐107 inhibits proliferation, migration, and invasion and promotes apoptosis of glioma cells. (a) Expression of miR‐107 in normal (*n* = 10) and glioma tissues (*n* = 23) examined by RT‐qPCR; (b) Expression of miR‐107 in normal brain glial cells (Heb) and in glioma cell lines (U251, A172, and T98G) examined by RT‐qPCR; (c) Expression of miR‐107 in A172 and T98G cells after miR‐107 mimic transfection examined by RT‐qPCR; (d) Proliferation of A172 and T98G cells determined by the EdU labeling assay; (e) Migration and (f) invasion abilities of A172 and T98G cells examined by the transwell assays; (g) Apoptosis rate of A172 and T98G cells determined by flow cytometry. Data were collected from three independent experiments and presented as mean ± SD. In (a), each spot indicates a sample; differences were compared by unpaired *t* test (a, c, d, e, f, and g) or one‐way ANOVA (b), *p < .05 versus Normal/Heb/NC mimic

To examine the role of miR‐107 in glioma, we first detected the miR‐107 expression in the glioma tissues. As shown in Figure 1a, a poor‐expression profile of miR‐107 was detected in the glioma tissues versus normal tissues. Likewise, the expression of miR‐107 was reduced in glioma cell lines (U251, A172 and T98G) compared to the normal Heb cells (Figure 1b). Among the glioma cell lines, A172 and T98G cells with relative low expression of miR‐107 were selected for subsequent use.

Artificial overexpression of miR‐107 was introduced into A172 and T98G cells via administration of LV‐carried miR‐107 mimic, and successful transfection was confirmed by RT‐qPCR (Figure 1c). Thereafter, the EdU labeling assay suggested that the DNA replication, namely, the proliferation ability of cells, was significantly reduced after miR‐107 upregulation (Figure 1d). The transwell assay results showed that the migration and invasion abilities of both A172 and T98G cells were suppressed by miR‐107 mimic versus mimic NC (Figure 1e,f). In addition, the apoptosis of A172 and T98G cells, according to the flow cytometry, was significantly reduced after miR‐107 overexpression (Figure 1g).

### 3.2 E2F1 suppresses miR‐107 transcription

**FIGURE 2:**
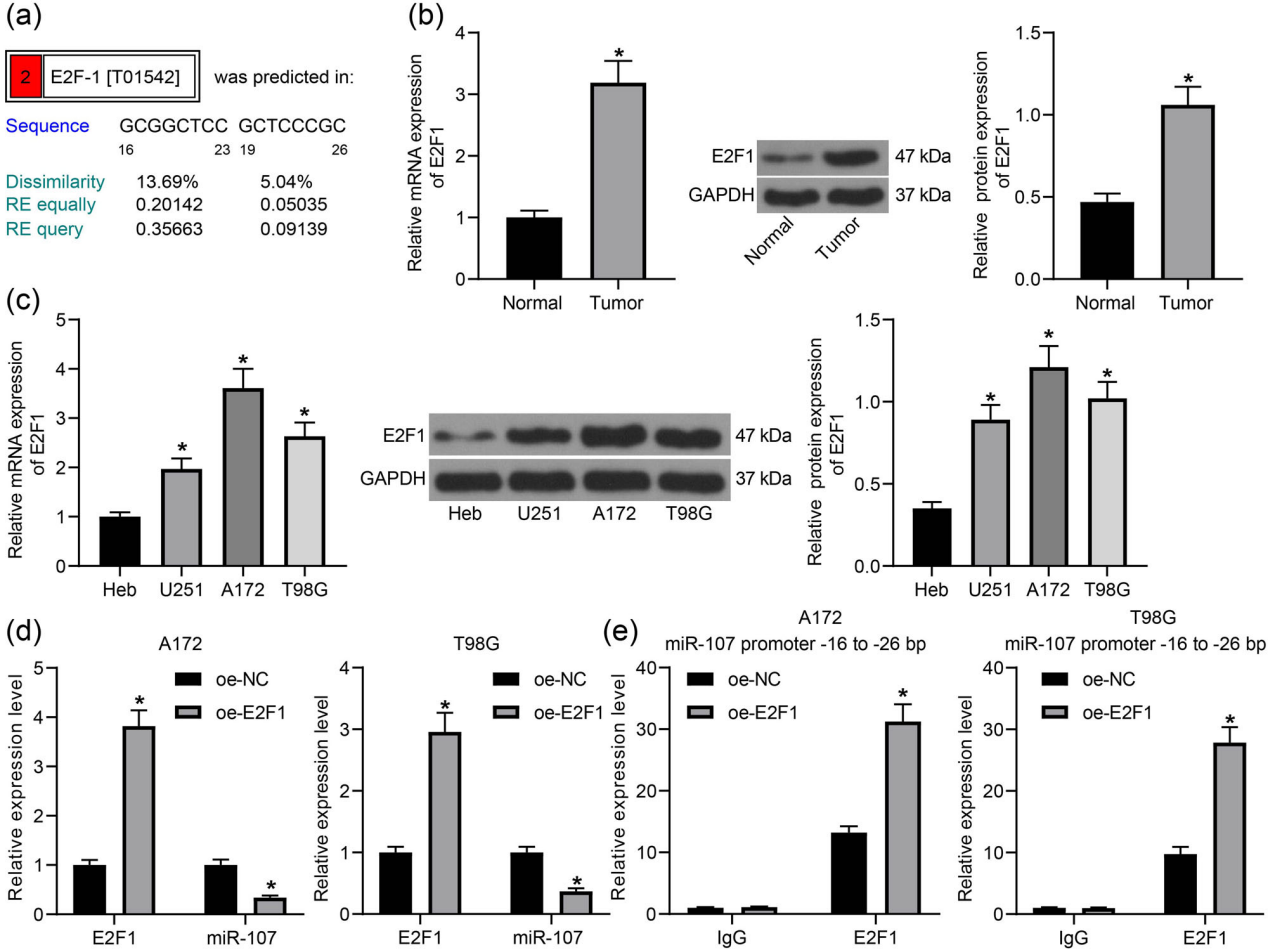
E2F1 suppresses miR‐107 transcription. (a) Potential binding sites between E2F1 and the promoter region of miR‐107 (−16 to −23 bp and −19 to −26 bp) predicted on the ALGEEN system; (b) mRNA and protein levels of E2F1 in glioma tumor tissues and in normal tissues determined by RT‐qPCR and western blot analysis, respectively; (c) mRNA and protein levels of E2F1 in normal brain glial cells (Heb) and in glioma cell lines (U251, A172 and T98G) examined by RT‐qPCR and western blot analysis, respectively; (d) Expression of E2F1 mRNA and miR‐107 in A172 and T98G cells after E2F1 overexpression examined by RT‐qPCR; (e) enrichment of E2F1 at the −16 to −26 bp site at the promoter region examined by a ChIP‐qPCR assay. Data were collected from three independent experiments and presented as mean ± SD. Differences were compared by unpaired *t* test (b), one‐way ANOVA (c), or two‐way ANOVA (d,e), *p < .05 versus Normal/Heb/oe‐NC

E2F1 has been reported as a cancer driver in glioma (Zhi et al., 2019). The promoter sequence of miR‐107 was obtained from UCSC (https://genome.ucsc.edu/index.html). Intriguingly, E2F1 was predicted to own a binding relationship with the promoter region of miR‐107 at the −16 to −26 bp site according to the ALGGEN system (http://alggen.lsi.upc.es/cgi‐bin/promo_v3/promo) (Figure 2a). Therefore, we examined the expression of E2F1 in glioma tissues and normal brain tissues. It was found that the mRNA and protein expression of E2F1 was higher in tumor tissues than in normal tissues (Figure 2b). In agreement with this, increased expression of E2F1 was found in U251, A172, and T98G cells compared to the Heb cells (Figure 2c).

Overexpression of E2F1 was introduced in A172 and T98G cells, after which the miR‐107 expression was significantly decreased (Figure 2d). The binding relationship between E2F1 and miR‐107 was validated via a chromatin immunoprecipitation (ChIP)‐qPCR assay. Importantly, an enrichment of E2F1 fragments was found at the −16 to −26 bp site at the promoter region of miR‐107 (Figure 2e). These results indicated that E2F1 can bind to the promoter region of miR‐107 to repress its transcription in glioma cells.

### 3.3 miR‐107 targets CCND1 mRNA

**FIGURE 3:**
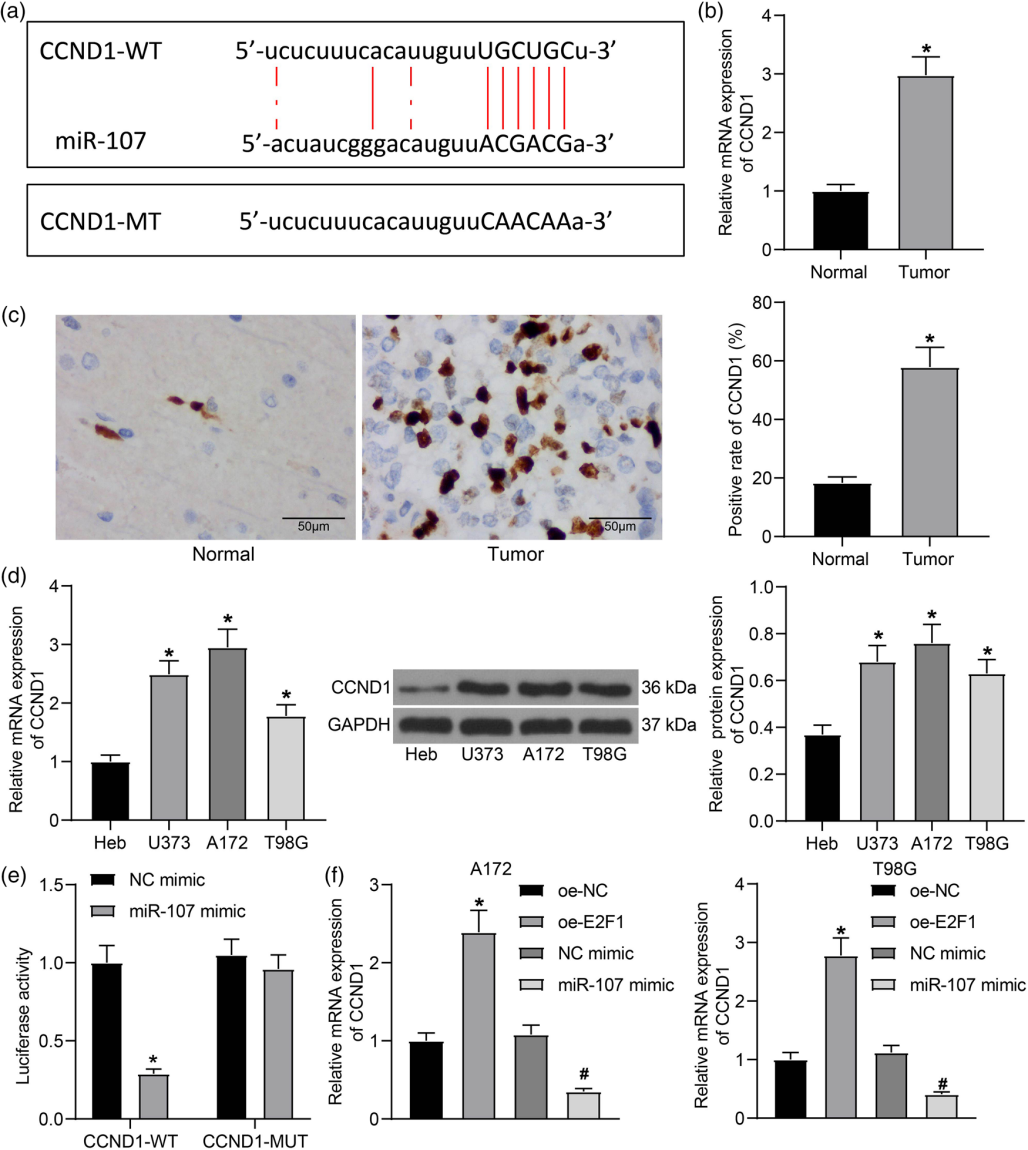
miR‐107 targets CCND1 mRNA. (a) Binding sequence between miR‐107 and CCND1 mRNA predicted on the StarBase system; (b) mRNA and (c) protein levels of CCND1 in normal brain tissues (*n* = 10) and glioma tissues (*n* = 23) examined by RT‐qPCR and IHC staining, respectively; (d) mRNA and protein levels of CCND1 in normal brain glial cells (Heb) and in glioma cell lines (U251, A172, and T98G) examined by RT‐qPCR and western blot analysis, respectively; (e) Binding relationship between miR‐107 and CCND1 mRNA validated through a luciferase assay; (f) mRNA expression of CCND1 in A172 and T98G cells after oe‐E2F1 or miR‐107 mimic transfection examined by RT‐qPCR. Data were collected from three independent experiments and presented as mean ± SD. Differences were compared by unpaired *t* test (b and c), one‐way ANOVA (d and f), or two‐way ANOVA (e), *p < .05 versus Normal/Heb/oe‐NC; #*p* < .05 versus NC mimic

According to the bioinformatic analysis in the StarBase system (http://starbase.sysu.edu.cn/), miR‐107 was predicted to have a specific binding site with CCND1 mRNA (Figure 3a), which has been recently reported to be upregulated in glioma (Sun et al., 2020). We surmised that miR‐107 possibly regulates CCND1 expression to mediate glioma progression.

Therefore, we examined the expression of CCND1 in glioma samples. The RT‐qPCR and immunochemistry (IHC) staining results indicated that the level of CCND1 was higher in glioma tissues than in normal tissues (Figure 3b,c). In cells, the RT‐qPCR and western blot assays suggested that the mRNA and protein expression of CCND1 were higher in glioma cell lines (U251, A172, T98G) than in Heb cells (Figure 3d).

The binding relationship between miR‐107 and CCND1 mRNA was validated using a luciferase assay (Figure 3e). It was found that miR‐107 mimic significantly reduced the luciferase activity of CCND1‐3ʹUTR‐WT vector in 293T cells, whereas it had no effect on the activity of the CCND1‐3ʹUTR‐MUT luciferase vector.

The RT‐qPCR results also indicated that the level of CCND1 mRNA was increased in A172 and T98G cells in the setting of E2F1 overexpression. In addition, compared to NC mimic, transfection of miR‐107 mimic significantly reduced the CCND1 expression in the glioma cells (Figure 3f).

### 3.4 E2F1 regulates the miR‐107/CCND1 axis to promote malignant behaviors of glioma cells

**FIGURE 4:**
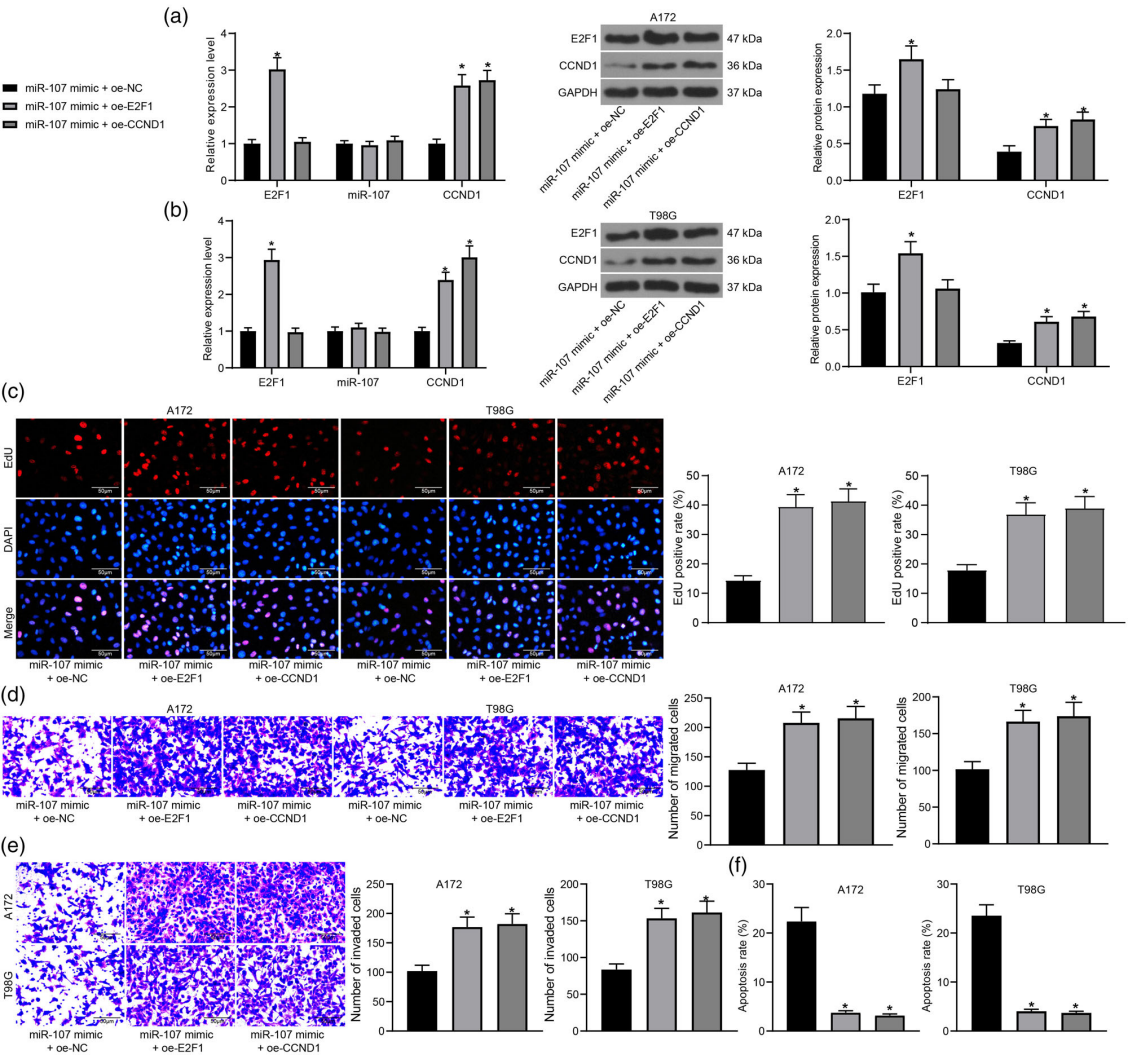
E2F1 regulates the miR‐107/CCND1 axis to promote malignant behaviors of glioma cells. Expression of miR‐107, E2F1 and CCND1 mRNA, and E2F1 and CCND1 protein in (a) A172 and (b) T98G cells determined by RT‐qPCR and western blot analysis, respectively; (c) Proliferation of A172 and T98G cells determined by the EdU labeling assay; (d) Migration and (e) invasion abilities of A172 and T98G cells examined by the transwell assays; (f) Apoptosis rate of A172 and T98G cells determined by flow cytometry. Data were collected from three independent experiments and presented as mean ± SD. Differences were compared by one‐way (c–f) or two‐way ANOVA (a,b), *p < .05 versus miR‐107 mimic +oe‐NC

To further validate the interactions between E2F1, miR‐107, and CCND1, the A172 and T98G cells were concomitantly transfected with miR‐107 mimic and oe‐E2F1 or oe‐CCND1. Importantly, overexpression of E2F1 reduced the expression of miR‐107 but increased the level of CCND1 in A172 and T98G cells. Concomitant transfection of oe‐CCND1 did not alter the expression of E2F1 or miR‐107, but only increased the expression of CCND1 in cells (Figure 4a,b).

The malignant behaviors of cells with miR‐107 mimic and oe‐E2F1/oe‐CCND1 transfections were examined. The EdU labeling assay results suggested that the proliferation ability of A172 and T98G cells inhibited by miR‐107 mimic was restored upon E2F1 or CCND1 overexpression (Figure 4c). Also, the migration and invasion potentials of cells were recovered by oe‐E2F1 or oe‐CCND1 according to the transwell assays (Figure 4d,e). Moreover, the flow cytometry results indicated that the miR‐107‐induced apoptosis in A172 and T98G cells was blocked after E2F1 or CCND1 upregulation (Figure 4f).

### 3.5 E2F1 mediates the Wnt/β‐catenin signaling pathway

**FIGURE 5:**
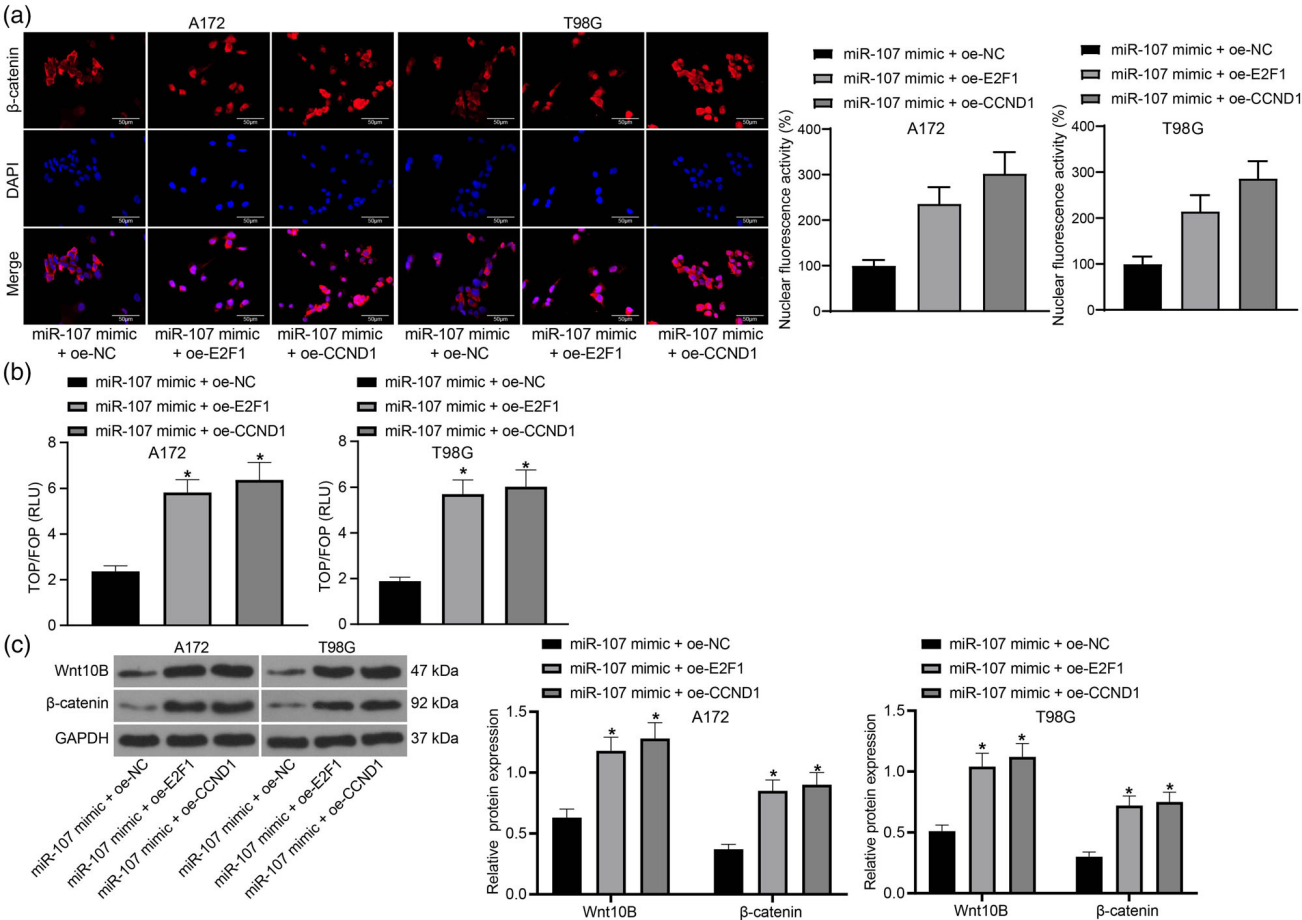
E2F1 mediates the Wnt/β‐catenin signaling pathway. (a) Nuclear translocation of β‐catenin in A172 and T98G cells examined by immunofluorescence staining; (b) TOP/FOP activity in A172 and T98G cells determined by the TOP/FOP flash assay; (c) Protein levels of Wnt10B and β‐catenin in A172 and T98G cells detected by western blot analysis. Data were collected from three independent experiments and presented as mean ± SD. Differences were compared by one‐way ANOVA (b) or two‐way ANOVA (c), *p < .05 versus miR‐107 mimic +oe‐NC

CCND1 is one of the downstream targets of the Wnt/β‐catenin pathway, but it can also regulate the nuclear translocation of β‐catenin (Xia et al., 2019). Here, we examined the nuclear translocation of β‐catenin using immunofluorescence staining and TOP/FOP activity using the TOP/FOP flash assay. As shown in Figure 5a,b, either overexpression of E2F1 or CCND1 increased the nuclear accumulation of β‐catenin as well as the activity of TOP/FOP in A172 and T98G cells.

The nuclear levels of the Wnt/β‐catenin signaling‐related proteins Wnt10B and β‐catenin were further examined by western blot analysis. In concert with the above results, increased nuclear protein levels of Wnt10B and β‐catenin were detected in A172 and T98G cells transfected with miR‐107 mimic + oe‐E2F1 or miR‐107 mimic + oe‐CCND1 (Figure 5c).

### 3.6 The functions of E2F1, miR‐107, and CCND1 in tumorigenesis of glioma cells in vivo

**FIGURE 6:**
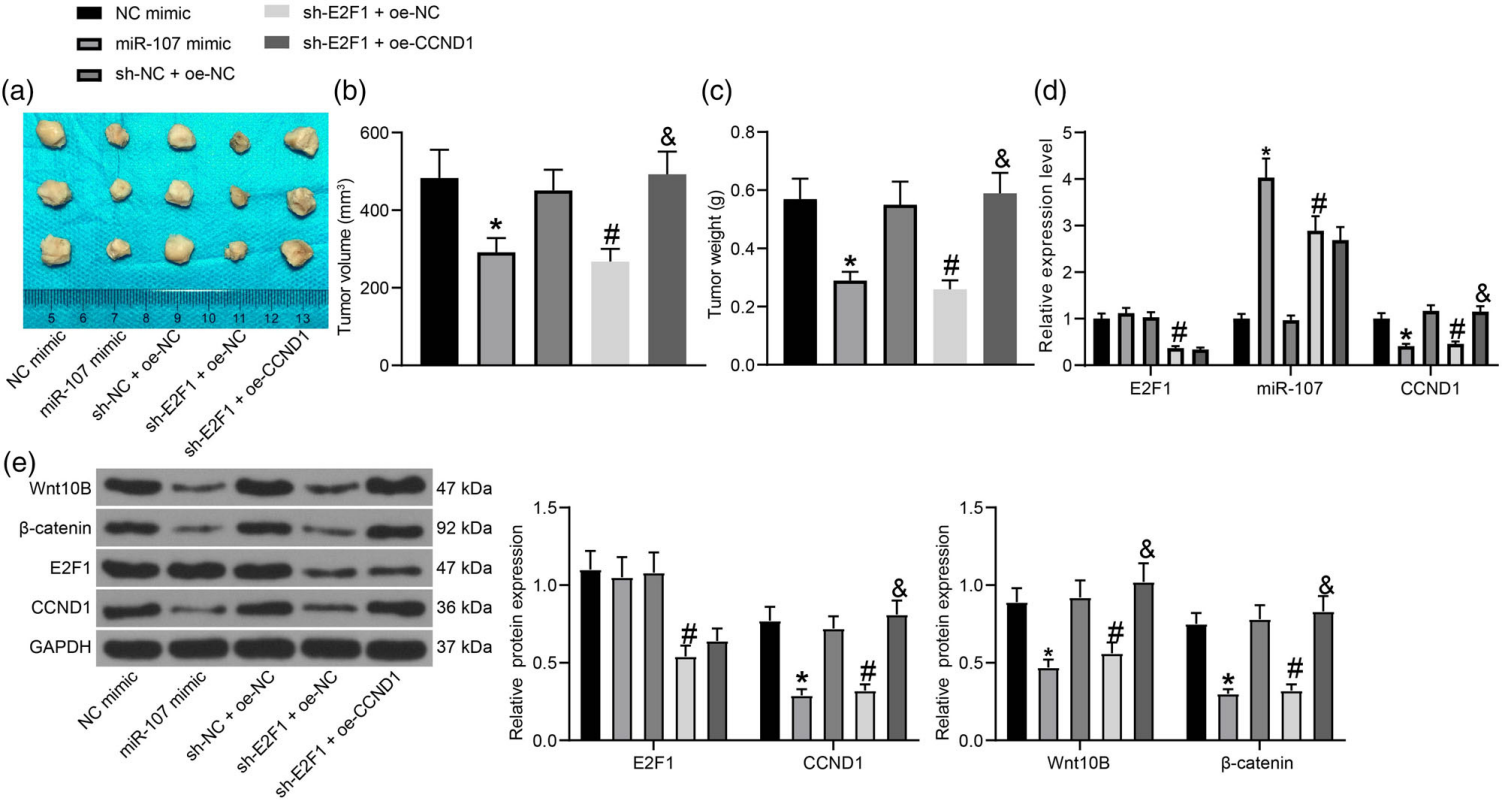
The functions of E2F1, miR‐107 and CCND1 in tumorigenesis of glioma cells in vivo. (a) Images of the tumors in each group; (b) Volume and (c) weight of the xenograft tumors on the 21st day after cell injection; (d) Expression of miR‐107 and E2F1 and CCND1 mRNA in tumor tissues examined by RT‐qPCR; (e) Protein levels of E2F1, CCND1, Wnt10B, and β‐catenin in tumor tissues examined by western blot analysis. Data were collected from three independent experiments and presented as mean ± SD. In each group, *n* = 5. Differences were compared by one‐way ANOVA (b,c) or two‐way ANOVA (d,e), *p < .05 versus NC mimic; #*p* < .05 versus sh‐NC + oe‐NC; and *p* < .05 versus sh‐E2F1 + oe‐NC

To further examine the role of E2F1/miR‐107/CCND1 axis in glioma progression, A172 cells stably transfected with NC mimic, miR‐107 mimic, sh‐NC + oe‐NC, sh‐E2F1 + oe‐NC and sh‐E2F1 + oe‐CCND1 were injected into nude mice for in vivo validation. Importantly, either transfection of miR‐107 mimic or sh‐E2F1 significantly reduced the volume and weight of the xenograft tumors formed by A172 cells in mice. However, overexpression of CCND1 in A172 cells restored the growth rate and weight of the xenograft tumors which was suppressed by sh‐E2F1 (Figure 6a–c).

The expression of miR‐107, E2F1, and CCND1 in the tumor tissues was examined. First, the RT‐qPCR results showed that miR‐107 mimic reduced the expression of CCND1 in the tumor tissues. Compared to sh‐NC + oe‐NC, transfection of sh‐E2F1 + oe‐NC in the cells reduced the expression of E2F1 and CCND1 in the tumor tissues. Transfection of oe‐CCND1 in the cells increased CCND1 expression in tissues, whereas it did not alter the expression of E2F1 or miR‐107 (Figure 6d,e). The tumor tissues were further collected for western blot analysis. The protein levels of Wnt10B and β‐catenin in tumor tissues were reduced by miR‐107 mimic or sh‐E2F1. However, further transfection of oe‐CCND1 in tissues blocked the function of sh‐E2F1 and restored the protein levels of Wnt10B and β‐catenin (Figure 6e).

## 4 DISCUSSION

Gliomas, which represent 80% of all malignant tumors in the CNS, are virtually incurable since the 5‐year overall survival rate of the most common but aggressive type GBM is no more than 5%, even following the optimal treating combinations (Chen et al., 2012; Hombach‐Klonisch et al., 2018). Researchers in this field have made significant efforts in identifying the molecular mechanisms involved in cancer progression. Here, this study reports that there might be an E2F1/miR‐107/CCND1 axis that mediates the malignant development of glioma cells in vitro and in vivo with the implication of Wnt/β‐catenin signaling.

MiRNAs have emerged as key molecules having important diagnostic and prognostic values in glioma (Mondal & Kulshreshtha, 2021; Wang et al., 2019). Recent studies mainly focused on their interactions with other ncRNAs (Wu & Qian, 2019). miR‐107 has been demonstrated as a tumor expressed at low levels in several human cancers such as colorectal cancer (Fu et al., 2019), cervical cancer (Li et al., 2019; Rui et al., 2018), and non‐small cell lung cancer (Fan et al., 2020). However, studies have also suggested that miR‐107 may confer chemoresistance to cancer cells (Liang et al., 2020) and promote growth and invasiveness of gastric cancer (Wang et al., 2019). This may be attributed to the different genes they regulated. In this study, we observed that miR‐107 expression was reduced in glioma tissues and the acquired glioma cells. Artificial upregulation of miR‐107 reduced proliferation, migration and invasion, and increased apoptosis of A172 and T98G cells. In concert with this, poor expression of miR‐107 has been found in glioma in previous reports (Su & Song, 2018; Zhen et al., 2019). Restoration of miR‐107 facilitated apoptosis, increased chemo sensitivity, and weakened proliferation, invasiveness, and angiogenesis of glioma cells (Chen et al., 2016; Su & Song, 2018; Wu et al., 2020). In the study, a similar trend was reproduced in the in vivo experiments where upregulation of miR‐107 in A172 cells significantly reduced the volume and weight of xenograft tumors.

E2F1 has been reported as an oncogene, and its downregulation by miRNAs has been suggested to reduce malignant behaviors, such as proliferation, invasiveness, and resistance to apoptosis of cancer cells (Han et al., 2020; Lu et al., 2018; Peng et al., 2020). This is also true for glioma, because upregulation of E2F1 has been correlated with proliferation, cell cycle progression, and carcinogenesis of glioma (Li et al., 2019; Xia et al., 2019). In addition, previous studies have demonstrated that E2F1 can transcriptionally regulate multiple miRNAs, such as elevating miR‐17−92 cluster, miR‐15/16, miR‐203, and miR‐449a/b levels, and reducing miR‐30b and miR‐1205 levels (Li et al., 2019; Ofir et al., 2011; Tan et al., 2014; Wang et al., 2015; Yang et al., 2009; Zhang et al., 2015). E2F1 belongs to the E2Fs family of transcriptional factors which regulate a multitude of genes involved in multiple key cellular processes. Here, we predicted the potential binding site between E2F1 and the promoter region of miR‐107 and had the binding relationship validated using a ChIP‐qPCR assay. As expected, high expression of E2F1 was confirmed in the glioma tissues and cells, which showed an inverse trend with miR‐107. Importantly, the proliferation, migration and invasion, and the resistance to apoptosis of cells reduced by miR‐107 mimic were restored upon further overexpression of E2F1. These results suggested that downregulation of miR‐107 is possibly implicated in the oncogenic events mediated by E2F1 in glioma.

The bioinformatics analysis and luciferase assay confirmed CCND1 as a target transcript of miR‐107. CCND1 is a crucial cell cycle regulatory protein whose expression and cellular localization is frequently transformed in tumor cells (Xie et al., 2017), and it is capable of inducing cell proliferation, invasion, and transformation in human malignancies (Lin, 2017). This is also true for glioma (Alqudah et al., 2013; Yamada et al., 2018). Here, we found CCND1 was highly expressed in glioma tissues and cells, and its expression was reduced by miR‐107, but reduced by E2F1. Importantly, upregulation of CCND1 restored the proliferation, resistance to death, migration, and invasion of glioma cells and augmented the malignant growth of xenograft tumors. CCND1 is one of the important downstream targets of the Wnt/β‐catenin signaling, a master regulator in carcinogenesis (Ghanavati et al., 2020; Xia et al., 2019). Likewise, this signaling pathway is frequently activated during the pathogenesis of glioma (He et al., 2019). In the study by Xia et al. (2019), CCND1 has been reported to have elevated the nuclear translocation of β‐catenin in cells. The authors suggested that CCND1 can accelerate the β‐catenin of nuclear binding to the Nanog's promoter. As a transcriptional factor, Nanog possibly plays a role in the expression and nuclear accumulation of β‐catenin. However, the specific mechanism remains to be further explored. Interestingly, miR‐107 has been reported to suppress the activation of the Wnt/β‐catenin signaling pathway to suppress proliferation and metastasis of cancer (Yao et al., 2021; Yu et al., 2018). Here, we confirmed that miR‐107 reduces nuclear translocation of β‐catenin, whereas the activity of the Wnt/β‐catenin pathway was increased upon E2F1 or CCND1 overexpression.

## 5 CONCLUSION

**FIGURE 7:**
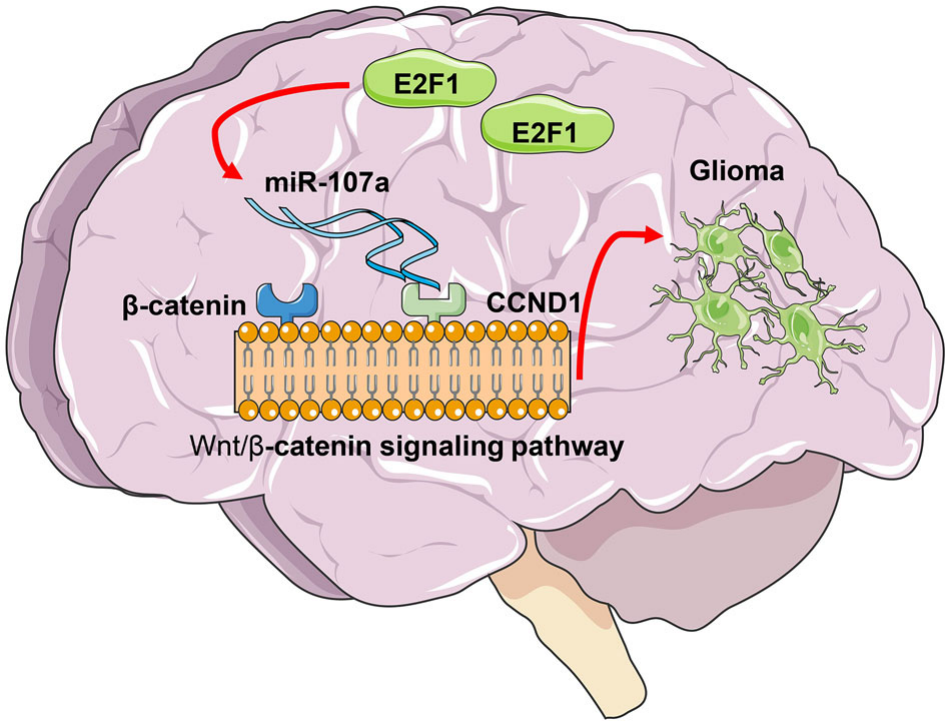
A graphic abstract. E2F1 binds to the promoter region of miR‐107 to suppress its transcription, which restores the expression of the miR‐107 target gene CCND1, therefore promoting growth and metastasis of glioma cells

In conclusion, by performing both cellular and animal experiments, we confirmed that the transcription factor E2P1 can induce transcriptional repression of miR‐107 and block its inhibitory effect on CCND1, which leads to the malignant development of glioma with the involvement of the Wnt/β‐catenin signaling pathway (Figure 7). This study confirmed the oncogenic roles of E2F1 and CCND1 and the anti‐tumorigenic role of miR‐107 in glioma. We hope these findings may offer a new understanding on the molecular mechanism involved in glioma pathogenesis.

## CONFLICT OF INTEREST

The authors declare no conflict of interest.

### TRANSPARENT PEER REVIEW

The transparent peer review history for this article is available at https://publons.com/publon/10.1002/brb3.2399

## Data Availability

All the data generated or analyzed during this study are included in this published article.
